# Advances in Biomarker Discovery for Cardiovascular Disease

**DOI:** 10.3390/biomedicines14102144

**Published:** 2026-09-23

**Authors:** Qin Fu, Haijun Liu

**Affiliations:** 1 Proteomics and Metabolomics Facility, Institute of Biotechnology, Cornell University, Ithaca, NY 14850, USA; 2 David Geffen School of Medicine, University of California, Los Angeles, CA 90095-1766, USA

## 1. Introduction

Cardiovascular disease remains one of the leading causes of morbidity and mortality worldwide, representing a major global public health challenge. Despite substantial advances in prevention, diagnosis, and treatment, early detection and accurate risk stratification of cardiovascular disorders remain challenging due to the complex and multifactorial nature of disease development [1]. Traditional clinical indicators, including lipid profiles, blood pressure measurements, cardiac troponins, and natriuretic peptides, have significantly improved patient management; however, there remains an urgent need for novel biomarkers that can enable earlier diagnosis, improve prognostic prediction, monitor therapeutic responses, and facilitate personalized treatment strategies [2].

Recent advances in high-throughput molecular technologies have greatly expanded our ability to investigate cardiovascular pathophysiology at multiple biological levels. Genomics, transcriptomics, proteomics, metabolomics, and integrative multi-omics approaches have provided unprecedented opportunities to identify molecular signatures associated with cardiovascular disease initiation, progression, and therapeutic response [3]. In particular, mass spectrometry-based proteomics and metabolomics have emerged as powerful platforms for large-scale characterization of proteins, peptides, post-translational modifications (PTMs), and metabolites, enabling the discovery of clinically relevant biomarker candidates and novel therapeutic targets [4].

Proteins and their modifications play critical roles in cardiovascular homeostasis and disease progression. Alterations in protein abundance, phosphorylation, glycosylation, oxidation, lipidation, and other PTMs can reflect dynamic changes in cellular signaling, inflammation, metabolism, and tissue remodeling [5]. Advances in enrichment strategies, high-resolution mass spectrometry, and computational analysis have significantly improved the sensitivity and depth of proteomic investigations, allowing researchers to uncover previously inaccessible molecular mechanisms underlying cardiovascular disorders [6]. These discoveries provide new opportunities for developing biomarkers with improved specificity and biological relevance.

Beyond individual omics layers, integrated multi-omics approaches are increasingly recognized as essential strategies for understanding the complexity of cardiovascular diseases [7]. Combining molecular profiles with clinical characteristics, imaging data, and patient outcomes can improve biomarker validation and accelerate translation from discovery research into clinical applications [8]. Artificial intelligence and machine learning approaches further provide powerful tools for integrating large-scale datasets and identifying predictive molecular signatures [9].

Nevertheless, several challenges remain before newly discovered biomarkers can be routinely implemented in clinical practice. These challenges include the reproducibility of biomarker measurements, standardization of analytical workflows, validation in large and diverse patient cohorts, and demonstration of clinical utility. Collaborative efforts among researchers, clinicians, and technology developers will be essential to overcome these barriers and advance promising biomarkers toward clinical translation.

## 2. An Overview of the Published Articles

The articles collected in this Special Issue provide complementary perspectives on the molecular, cellular, and clinical factors involved in biomarker identification and validation for cardiovascular disease. These studies highlight the complexity of cardiovascular pathophysiology and underscore the importance of integrating circulating biomarkers, inflammatory mediators, metabolic signatures, genetic factors, imaging parameters, and computational approaches to advance precision cardiovascular medicine.

A major theme emerging from this Special Issue is the identification of circulating molecular biomarkers associated with cardiovascular injury and disease outcomes. Mitochondrial dysfunction is increasingly recognized as a critical contributor to cardiovascular pathology. Peng et al. investigated the association between serum mitochondrial open reading frame of the 12S rRNA-c (MOTS-c) levels and myocardial ischemia–reperfusion injury (MIRI) in patients with acute myocardial infarction undergoing percutaneous coronary intervention. Their findings support the potential role of MOTS-c as a biomarker reflecting mitochondrial stress responses and myocardial injury, providing new insights into molecular mechanisms associated with ischemic cardiac damage [10].

Inflammation and immune dysregulation represent central mechanisms underlying cardiovascular disease progression. Several of the studies in this Special Issue focus on inflammatory biomarkers for cardiovascular risk assessment. Li et al. demonstrated that interferon gamma (IFN-γ) and tumor necrosis factor alpha (TNF-α) are associated with major adverse cardiovascular events (MACE) in patients with peripheral artery disease (PAD), suggesting their potential utility for identifying high-risk individuals [11]. In a complementary study, Li et al. evaluated circulating immunomodulatory proteins as predictors of MACE in PAD patients, further emphasizing the importance of immune-related molecular signatures in cardiovascular risk prediction [12]. Additionally, Akácsos-Szász et al. [13] investigated blood-count-derived biomarkers, homocysteine levels, and MTHFR genetic variants in patients with severe PAD, highlighting the potential value of combining accessible clinical parameters with molecular markers for disease assessment.

Metabolic pathways also play essential roles in cardiovascular disease development and prognosis. Kern et al. [14] investigated kynurenine, a metabolite generated through the tryptophan degradation pathway, as a long-term predictor of mortality using data from the KORONEF registry. Their findings highlight the contribution of metabolic alterations and inflammation-associated pathways to cardiovascular outcomes and support kynurenine as a potential prognostic biomarker.

Beyond molecular biomarkers, this Special Issue highlights the integration of biomarkers with functional measurements and clinical phenotypes. Chen et al. [15] investigated lysyl oxidase-like 2 (LOXL2) as a biomarker for guiding early cardiac rehabilitation in patients with acute heart failure. Their prospective cohort study demonstrated the potential of LOXL2-guided strategies for improving patient stratification and optimizing long-term cardiovascular care. Similarly, Teszak et al. [16] compared donor-derived cell-free DNA (dd-cfDNA) with left ventricular longitudinal strain and strain-derived myocardial work indices for identifying heart transplant injury, demonstrating the complementary value of molecular and imaging-based biomarkers for detecting cardiac damage.

Early detection of cardiovascular injury is particularly important in patients exposed to cardiotoxic therapies. Caballero-Valderrama et al. investigated myocardial strain alterations and miR-122-5p expression changes in patients receiving anthracycline treatment. Their study demonstrated that early reductions in global longitudinal strain-together with elevated miR-122-5p levels-were associated with interstitial fibrosis, supporting the use of integrated functional and molecular biomarkers for early detection of chemotherapy-induced cardiotoxicity [17].

This Special Issue also highlights the expanding role of non-coding RNAs and genetic variation in cardiovascular biomarker discovery. Stefanovic et al. [18] examined the expression profile of lncRNA MALAT1, miR-146a, and COX-2 six months after myocardial infarction, as well as the association of MALAT1 rs3200401 and miR-146a rs2910164 polymorphisms with disease susceptibility. This study provides further evidence that regulatory networks involving long non-coding RNAs, microRNAs, inflammatory pathways, and genetic variants may contribute to post-MI remodeling and represent promising biomarker candidates for cardiovascular risk assessment.

Computational and systems biology approaches are increasingly important for uncovering complex molecular signatures associated with cardiovascular disorders. Liu et al. [19] applied integrated bioinformatics approaches to identify programmed cell death-related targets associated with Pueraria-mediated protection in septic cardiomyopathy and constructed a diagnostic model. This study demonstrates the potential of combining molecular network analysis and computational modeling to identify novel biomarkers and therapeutic targets in complex cardiovascular conditions.

In addition to disease-associated molecular signatures, understanding vascular physiology and developmental cardiovascular disorders provides new opportunities for biomarker discovery. Caetano et al. [20] investigated homeostatic responses to subsystolic arterial occlusive pressure in glabrous and non-glabrous skin circulation, providing new insights into the systemic regulation of microvascular function and vascular biomarkers. Furthermore, Carauleanu et al. [21] reviewed emerging biomarkers detectable in maternal blood for early detection of fetal septal defects, highlighting the potential of minimally invasive biomarker strategies for improving prenatal cardiovascular diagnosis.

Collectively, the contributions in this Special Issue demonstrate the rapidly evolving field of cardiovascular biomarker discovery, moving beyond single-marker approaches toward integrated molecular and clinical frameworks. These studies encompass diverse biomarker classes, including mitochondrial peptides, inflammatory cytokines, metabolic intermediates, circulating nucleic acids, genetic variants, imaging-derived parameters, and computationally predicted molecular signatures. Together, they emphasize that successful biomarker translation requires rigorous validation, integration with clinical characteristics, and demonstration of clinical utility. Continued advances in multi-omics technologies, artificial intelligence, and precision medicine approaches will further accelerate the development of robust biomarkers for cardiovascular disease prevention, diagnosis, prognosis, and personalized treatment.

## 3. Conclusions

This Special Issue, entitled “Advances in Biomarker Discovery for Cardiovascular Disease” highlights recent progress in biomarker identification and validation across multiple molecular disciplines. The collected articles provide valuable insights into emerging biomarkers, innovative analytical approaches, and translational strategies aimed at improving cardiovascular disease diagnosis, prognosis, and treatment. Contributions in this Special Issue encompass diverse areas, including proteomics, metabolomics, inflammatory biomarkers, molecular mechanisms of cardiovascular injury, and novel approaches for precision medicine.

We sincerely thank all authors who contributed their valuable research and perspectives to this Special Issue. We also greatly appreciate the reviewers for their time, expertise, and constructive comments that helped ensure the quality of the published work. We hope that this Special Issue will stimulate further discoveries and collaborations, ultimately accelerating the development of innovative biomarker-based strategies for cardiovascular disease prevention, diagnosis, and personalized therapy.
